# Application of ATAC-seq in tumor-specific T cell exhaustion

**DOI:** 10.1038/s41417-022-00495-w

**Published:** 2022-07-06

**Authors:** Chufeng Chen, Jiaying Liu, Yidong Chen, Anqi Lin, Weiming Mou, Lingxuan Zhu, Tao Yang, Quan Cheng, Jian Zhang, Peng Luo

**Affiliations:** 1 Department of Oncology, Zhujiang Hospital, Southern Medical University, Guangzhou, Guangdong China; 2 Nanfang Hospital, Southern Medical University, Guangzhou, Guangdong China; 3 Department of Neurosurgery, Xiangya Hospital, Central South University, Changsha, Hunan China

**Keywords:** Innate lymphoid cells, Cell biology, Gene regulation

## Abstract

Researches show that chronic viral infection and persistent antigen and/or inflammatory signal exposure in cancer causes the functional status of T cells to be altered, mainly by major changes in the epigenetic and metabolic environment, which then leads to T cell exhaustion. The discovery of the immune checkpoint pathway is an important milestone in understanding and reversing T cell exhaustion. Antibodies targeting these pathways have shown superior ability to reverse T cell exhaustion. However, there are still some limitations in immune checkpoint blocking therapy, such as the short-term nature of therapeutic effects and high individual heterogeneity. Assay for transposase-accessible chromatin with sequencing(ATAC-seq) is a method used to analyze the accessibility of whole-genome chromatin. It uses hyperactive Tn5 transposase to assess chromatin accessibility. Recently, a growing number of studies have reported that ATAC-seq can be used to characterize the dynamic changes of epigenetics in the process of T cell exhaustion. It has been determined that immune checkpoint blocking can only temporarily restore the function of exhausted T cells because of an irreversible change in the epigenetics of exhausted T cells. In this study, we review the latest developments, which provide a clearer molecular understanding of T cell exhaustion, reveal potential new therapeutic targets for persistent viral infection and cancer, and provide new insights for designing effective immunotherapy for treating cancer and chronic infection.

## Introduction

CD8+ T cells play a crucial role in resisting viral and bacterial infection as well as anti-tumor [1, 2]. After T cells are exposed to chronic infection or tumor, they can become exhausted and dysfunctional [3]. The increased and persistent expression of inhibitory receptors (IRs), such as programmed death 1 (PD-1) [4], lymphocyte activation gene 3 (LAG3) [5], T cell immunoglobulin and mucin domain 3 (TIM-3), and cytotoxic T lymphocyte-associated protein 4 (CTLA-4) [4] are one of the key features of exhausted CD8+ T cells (Tex). In addition, they gradually lose their effector functions, change epigenetic and transcriptional profiles, change their metabolic patterns, slow down their proliferation, and respond slowly to stimuli [6]. This is caused by CD8+ T cells being deficient in cytokines (IL-2, TNF, and IFN-γ).

Through the deep application of gene chips, also known as DNA microarrays, and RNA sequencing technology, scientists have obtained a clearer view of the genomic and transcriptome atlases of Tex [3]. Despite such advancements, researchers still know less about the epigenetic characteristics of Tex. Therefore, it remains vital to explore the molecular dynamic changes during the exhaustion of T cells from the epigenetic level in order to have a macroscopic understanding of the whole process of Tex.

Understanding the underlying mechanism for blocking immune checkpoints and finding new targets are critical scientific problems that should be solved in order to restore the function of Tex to improve efficacy of immune checkpoints inhibitor therapy [7]. Therefore, a deeper understanding of the dynamic changes of the epigenetic characteristics of T cell exhaustion is indispensable. In this review, we focus on the changes of Tex chromatin accessibility and epigenetic characteristics after blocking immune checkpoints characterized by ATAC-seq. Moreover, we present a new and more comprehensive view on the potential regulatory mechanism of T cell exhaustion in epigenetics, especially regarding the complex interaction between different transcription factors. In addition, we emphasize the therapeutic targets that can be used to restore the effect function of T cells. Finally, we explain that the reason for immune checkpoint blocking being able to only temporarily restore the function of Tex lies in its irreversible changes of epigenetics.

## ATAC-seq provides a new perspective for studying the occurrence and progression of Tex

### ATAC-seq helps characterize epigenetic features of Tex

**Fig. 1:**
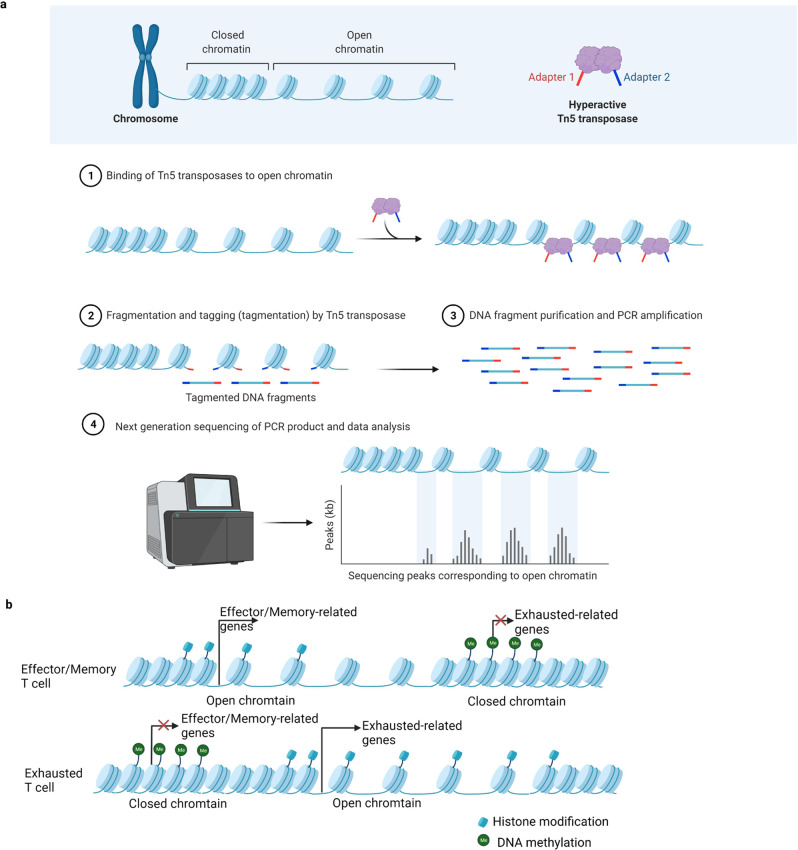
ATAC-seq helps characterize epigenetic features of Tex. **a** The principle and process of ATAC-seq. **b** Transcription factors affect the potential mechanism of chromatin landscape change through DNA methylation, histone modification and overall epigenetic regulation.

ATAC-seq offers the ability to analyze the accessibility of whole-genome chromatin (Fig. 1a). It uses hyperactive Tn5 transposase to assess chromatin accessibility [8]. ATAC-seq technology marks the open chromatin by inserting sequence adapters at both ends of Tn5 DNA transposase, thus obtaining information such as the position of the open chromatin, the binding site of transcription factors, the regulatory region of the nucleosome, and the chromatin state [8]. ATAC-seq makes it possible to acquire an abundance of epigenetic information, including open chromatin atlas, nucleosome localization, and the occupancy of transcription factors at a genomic locus. Transcription factors and regulatory regions can also be obtained by identifying transcription factor binding sites in open chromatin regions. ATAC-seq possesses several advantages compared with the traditional methods utilized in studying open chromatin, such as MNase-seq, DNase-seq, FAIRE-seq and ChIP-seq. One such advantage is the fewer numbers of cells that are required. Moreover, its shorter experimental period and simpler operation have made it the preferred technique for relevant research [8]. It is also beneficial to construct regulatory networks from DNA to RNA to phenotype, and to search for core regulatory factors strongly related to phenotypes. Since it has not been carried out for a long history, it also has some disadvantages, such as immature data analysis tools, contamination of generated data with mitochondrial DNA, and it requires 60 to 100 million reads for standard accessibility studies of the human genome [9, 10]. ATAC-seq is usually combined with RNA-seq, ChIP-seq, Hi-C, and other technologies to study the regulation mechanism of gene expression [11] and draw chromatin accessibility atlases of human primary tumors and other diseases [12–14]. Thus, it reveals the changes in the regulatory regions that drive the occurrence and progression of disease and promotes the study of complex diseases at the epigenetic level. Therefore, the application of ATAC-seq will help characterize epigenetic features of Tex.

### Comparison of the chromatin accessibility between tumor-specific Tex and chronic viral infection-specific Tex

Common characteristics can be seen in the CD8+ T cell function of patients suffering from chronic infections and tumors. In both cases, the expression of exhausted markers in CD8+ T cells increased and the dysfunctional CD8+ T cells transformed into exhausted cells due to stimulus of persistent antigens [15]. With the continued refinement of ATAC-seq technology and its convenience to explore chromatin accessibility in epigenetic research, some researchers have recently begun to explore whether the epigenetic state of specific exhausted CD8+ T cells caused by tumors and chronic infections is the same or similar. Defining the chromatin accessibility by ATAC-seq, two exhausted CD8+ T cell subsets from B16-OVA tumor and Lymphocytic choriomeningitis virus (LCMV) CI13 infection displayed the typical epigenetic characteristics, such as specific exhausted enhancers in the gene encoding Programmed cell death protein-1 (PD-1) and changes in chromatin-accessible regions (ChARs) around Tox. In another similar study, the researchers constructed high-resolution atlases of chromatin accessibility peaks of various types of T cells in mouse models, using acute bacterial infection, acute viral infection, and chronic viral infection [15]. In the PCA analysis of peaks in the atlas, the chromatin state of T cells infected by chronic virus and exhausted tumor-infiltrating lymphocytes (TILs) from different tumor models formed an obvious cluster [15]. The above studies show that although there are differences between cancer and chronic viral infection that lead to the distinct formation of the disease-specific epigenetic atlas, the chromatin accessibility characteristics of Tex observed by ATAC-seq are similar, even in the cross-tumor models. Exhausted CD8+ T cells in tumors and chronic viral infections share common epigenetic and transcriptional state space.

It can be seen that T cell exhaustion represents the basic adaptation process to chronic antigen stimulus and inflammatory conditions, and there is a common procedure across models and immune states. In addition, compared with chronic infection, exhausted TIL presents unique and subtle epigenetic changes, which may reflect the unique microenvironment of cancer. This includes changes in metabolism and cytokine signaling, which may fine-tune the basic process of T cell exhaustion [16].

### Comparison of ChARs among Tex, Teff, and Tmem

**Fig. 2:**
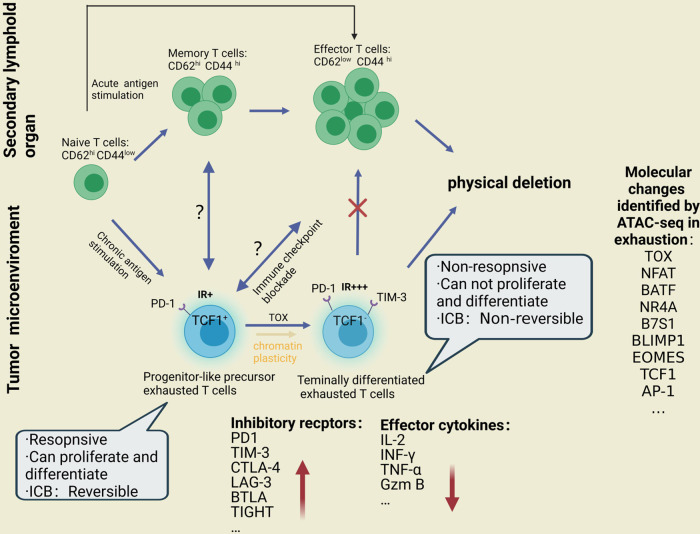
The potential developmental trajectory from progenitor Tex to terminal Tex and its comparison and relationship with Teff and Tmem. Tex possesses a unique epigenetic landscape compared with naive T cells, Teff and Tmem. Besides, Tex shows different regulatory processes in different stages, such as inhibitory receptors, effector cytokines, and motifs.

Recent evidence has been presented, showing that Tex (Exhausted CD8+ T cells) may be accompanied by the specific chromatin accessibility changes of CD8+ T cells and possesses a unique epigenetic landscape compared with naive T cells, Teff (Effector CD8+ T cells), and Tmem (Memory CD8+ T cells) [7, 17–20] (Fig. 2). Studies have noted that Tex have about 6000 different ChARs compared with Teff and Tmem [21], the researchers identified all the accessible regions of the genome in Tex and Teff genomes through the ATAC-seq technique, thus revealing the significant differences in regulatory region patterns: the ChARs of their gene expression component are significantly more than their differentially expressed genes [17]. Furthermore, a trajectory analysis based on ATAC-seq data revealed that EOMES and TBX21 (T-bet) motifs play a key role in the differentiation of naive CD8+ T cells into Teff or Tmem [22–25], proving the accessibility change of other known regulatory sites (including TFAP4 and YY1) [26, 27].

In contrast, Tex shows a unique regulatory process that features two stages. The first stage (intermediate Tex) mainly shows that changes in the accessibility, such as inhibitory receptors, tissue retention-related genes (such as ITGAE (CD103) [28] as well as motifs (such as NR3C1, NR4A1, and RUNX3 [29–32] can induce Tex. The second stage (terminal Tex) presents the accessibility of cis-elements near genes related to terminal Tex, such as CD101 and TOX [7, 19, 33–35]. More importantly, at this stage, there is a group of accessibility changes of TF motifs, including a basic leucine zipper ATF-like transcription factor (BATF), interferon regulatory factor 4 (IRF 4), nuclear factor kappa B subunit 1 (NFKB 1), Nuclear factor kappa B subunit 2 (NFKB 2) and nuclear factor of activated T cells (NFAT), which are located downstream of T-cell receptor (TCR) signal transduction. These have been proven to play a key role in Tex in mice [36–39]. This is the first time that researchers have observed the epigenetic characteristics of the differentiation track of exhausted TILs from naive CD8+ T cells to terminal Tex.

Additionally, in a follow-up study, researchers also mapped the regulatory elements of chromatin accessibility in naive, effective, memory, and exhausted CD8+ T cells of mice using ATAC-seq technology [18]. Notably, the research found that peaks appeared frequently in the promoter, intron and distant intergenic regions, and the sequences around the ATAC-seq peaks were highly conserved. However, Compared with effector or memory CD8+ T cells, cells with an exhausted phenotype were also characterized by the loss of accessibility in some regions, such as the intron peak in Satb1 locus, which is related to low Satb1 expression [19]. The “exhaustion-related” ChARs identified by ATAC-seq were used to predict which cellular proteins might be recruited to these sites to activate the exhaustion-specific gene expression programs, such as NFAT [40]. Interestingly, the regions reported in this study overlap with those noted in previous studies, which mapped as NFAT binding sites in T cells exposed to virus-infected cells over a long period [38, 40]. This may mean that the same pathway is used in cancer and chronic viral infection to induce T cells into an exhausted state. Further analysis of the Nr4a protein family, which was previously thought to have little association with Tex, strongly suggests that it in fact promotes T cell exhaustion [30, 41]. In addition, NFAT plays a complex role in T cells [40], indicating that DNA binding factors, such as NFAT and Nr4a protein, may jointly promote T cell dysfunction.

In summary, by locating the genome regulatory regions in Tex,Teff, and Tmem with ATAC-seq, it has been found that the epigenetic landscape of these regions is fundamentally different, and these types of cells control their gene activities through different pathways. Obviously, ATAC-seq technology provided a new perspective on studies of Tex [17, 42].

### Progenitor Tex and terminal Tex in tumor have different epigenetic and transcriptional states

A shift from a reversible state to an irreversible exhaustion state of CD8+ T cells in early-stage cancer. That is, two distinct states were described: progenitor exhausted CD8+ T cells (progenitor Tex) and terminally exhausted CD8+ T cells (terminal Tex) [19, 43–45]. Moreover, progenitor Tex and terminal Tex possess distinct epigenetic and transcriptional states (Supplementary Table 1).

Previously, TCF-1 was widely recognized as an important transcription factor for maintaining Tpro under the conditions of chronic viral infection and cancer [21, 45–48]. Moreover, BATF binds a large number of enhancer regions necessary for the differentiation of Tpro into Teff to regulate the expression of the desired genes. According to a model using chronic viral infection and CAR-T cells to treat solid tumors, BATF can block T cell exhaustion and guide Tpro to differentiate into Teff, rather than terminal Tex [49, 50]. Because of its high cellular plasticity, Tpro cells are considered the potential targets for cellular immunotherapy of tumors. Furthermore, anti-PD-L1 and other treatments can only enhance the self-replication of Tpro; thus, they cannot guide the differentiation of them into cytotoxic Teff [51]. Therefore, it is possible to block the differentiation of them into Tex and promote their differentiation into Teff by targeting BATF. This discovery provides a new approach for cellular immunotherapy, namely blocking the differentiation into Tex instead of reversing them.

## The potential transcriptional regulatory network of Tex supplemented by ATAC-seq

**Fig. 3:**
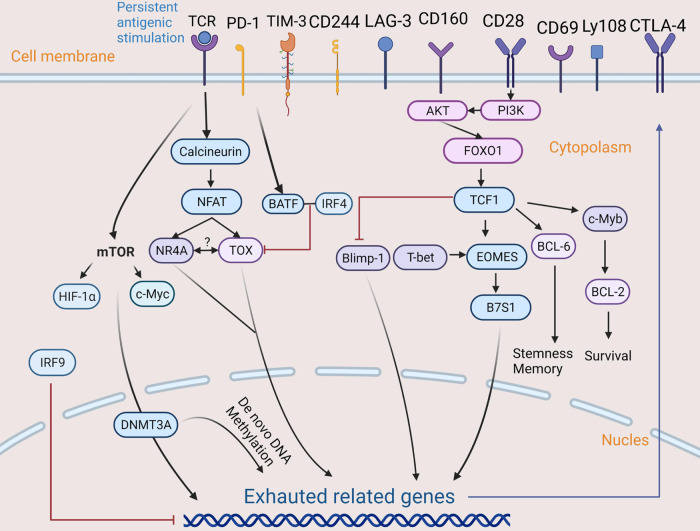
The complex regulatory network of Tex. The chronic TCR signal is the core driver of exhaustion, which activates NFAT and its downstream molecules (for example, TOX, NR4A and IRF4) through calcineurin, thereby up-regulating the expression of inhibitory receptors and maintaining the survival of T cells. In the absence of transcription factor AP-1, NFAT activates negative regulatory programs in CD8+ T cells and induces low reactivity in T cells. Then, activation of the secondary transcription factors TOX and NR4A will initiate the CD8+ Tex transcription process. TOX promotes and maintains Tex, while NR4A induces PD-1 and TIM-3 expression and weakens the anti-tumor effect of T cells. BATF is a downstream molecule of PD-1, which can inbibit T cells proliferation, cytokine secretion, and TOX when it combines with IRF4. In addition, transcription factor TCF1 is helpful for maintaining the stem cell-like characteristics of TILs and inducing the transformation of progenitor exhausted T cells. The PI3K/AKT/FOXO1 signaling network is activated by costimulatory molecule CD28. FOXO1 promotes the expression of TCF1, which mediates the transformation of T-bet-to-Eomes transcription factors by promoting Eomes expression in progenitor exhausted CD8+ T cells. TCF1 can also promote the expression of BCL-6 and c-Myb. The latter controls the survival and expression of BCL-2. The overexpression of Eomes induces Tex by activating B7S1. It is worth noting that Blimp-1, like T-bet, can also promote Tex, but Blimp-1 is inhibited by TCF-1. In addition, IRF9 may inhibit the exhaustion of TILs. As for metabolism, mTOR regulates the metabolic checkpoint of glycolysis through transcription factors HIF-1α and c-Myc and participates in methylation-specific exhausted procedures through epigenetic enzymes such as DNMT3A. Moreover, the de novo DNA methyltransferase DNMT3A induces epigenetic silencing of effector and memory-related genes by de novo DNA methylation.

There is an interaction of transcription factors (TFs) in Tex in cancer. The interaction can affect chromatin structure and gene expression levels, resulting in failure of cell differentiation along predefined lineage pathways, which leads to exhaustion. Ascertaining the interaction between chromatin accessibility and transcription factor binding is the basis for understanding transcription regulation and exhausted phenotype establishment of Tex [52]. In addition, the change of the transcription program is not only affected by TFs near the transcriptional start sites (TSSs), but also controlled by epigenetic changes of various DNA and histone modifications of the whole-genome regulatory elements [53] (Fig. 1b). At present, the development of ATAC-seq technology and its application in the field has greatly facilitated researches. It allows researchers to identify TFs related to exhaustion and the low response state of Tex, and to explore potential transcriptional regulation network (Fig. 3).

### TOX is the key inducer of T cell exhaustion

Recently, four studies have collectively shed light on how thymocyte selection-associated high-mobility group box protein (TOX), as a key regulator, participates in the process of T cell exhaustion in chronic viral infection and cancer [33–35, 54]. TOX is a nuclear DNA binding protein and a member of the HMG high-mobility group box superfamily that binds to nuclear DNA in a structure-dependent manner, as opposed to being sequence-dependent [55]. TOX plays an important role in the development of CD4+ T cells, NK cells, and intrinsic lymphocytes in the thymus [56].

The gene TOX is differentially expressed among Teff, Tmem, and Tex, and its high expression in Tex is critical in inducing the exhausted T cells phenotype defined by marker proteins PD-1, TIM3, LAG3, TIGIT, and EOMES [57, 58]. The ATAC-seq results indicate that the chromatin accessibility of the TOX locus is increased in Tex compared with Teff [33]. These results suggest that TOX may play a key regulatory role in Tex differentiation. Furthermore, the studies unanimously found that the expression of TOX positively correlated with the expression of multiple inhibitory receptors and negatively correlated with the expression of TCF1; it also was positively regulated by NFAT [33–35, 54]. Moreover, TOX is crucial for regulating the formation of transcriptome and epigenetic group characteristics of antigen-specific Tex of LCMV, HCV, and the primary liver cancer model [35, 59]. It can induce CD8+ T cells to take on the main characteristics of Tex and is not involved in the formation process of memory T cells or Teff. In addition, although the CD8+ T cells after TOX knockout showed a strong effector function in the early stage, indicating that the expression of inhibitory receptors, such as PD-1, decreased, while the number of T cells decreased greatly in the later stage [33–35]. The studies mentioned above combined ATAC-seq and multi-omics methods to verify the key role of TOX in the normal function of T cells and the survival of Tex. TOX acts as an important molecule that specifically regulates the differentiation of dysfunctional T cells, which can initiate and dominate the differentiation of Tex from the transcription level and epigenetics level. These studies have also discovered the specific pathways of upstream and downstream regulation of TOX in Tex.

Furthermore, TOX also combines with proteins involved in inhibitory epigenetic events, including DNMT1, LEO1, PAF1, SAP130, and SIN3A, which characterize the interactions with proteins involved in chromatin opening and closure [33]. Therefore, TOX can bind to and possibly recruit many different types of chromatin remodeling proteins and may regulate chromatin accessibility and indirectly affect gene expression by changing the transcription factor network and its target in Tex, thus inducing the differentiation of Tex. This indicates that TOX, as a key inducer of typical characteristics of Tex, plays an important role in the specific epigenetic program of Tex.

The above results show that: First, the epigenetic changes of TOX and the corresponding molecular events are crucial for the development of T cell exhaustion and help identify an epigenetic programming mechanism for it. Tox might be used as a therapeutic marker. Second, based on ATAC-seq and multi-omics, TOX, a key molecule for regulating Tex, was discovered, indicating that similar methods can be used to explore targets for tumor immunotherapy in the future. Third, Tex is a different cell subgroup from Teff and Tmem with ATAC-seq providing the corresponding molecular mechanism support for this group differentiation.

### NFAT, the upstream target of TOX, is the key regulator of T cell exhaustion

NFAT is a key regulatory factor of Tex [60]. It has been shown that there is a significant increase in the accessibility of chromatin at the NFAT1 binding sites in Tex [18, 19, 38–40]. Comparing chromatin immunoprecipitation sequencing (ChIP-seq) data sets [38] and the ATAC-seq data sets [19, 34] reveals that NFAT1 binds to the region within the TOX locus and more regions of chromatin accessibility are opened significantly in Tex. Meanwhile, after inhibiting NFAT with calcineurin inhibitor FK506 [19, 61, 62], there was a decrease in the expression of TOX and PD-1 in Tex, while the level of TCF-1 was increased. This indicates that NFAT regulated TOX expression and influence the differentiation of Tex [34].

In addition, NFAT plays a significant role in the effector or exhausted reactions of CD8+ T cells initiated by TCR signals, and the balance between them depends on transcriptional partners of NFAT. For acute immune responses, NFAT mainly coordinated with its transcription factor partner activator protein-1 (AP-1) to induce effector T cells [38, 63]. Classic AP-1 is composed of a heterodimer of basic region leucine zipper (bZIP) transcription factors FOS and JUN [64]. FOS and JUN family members are expressed briefly and then quickly made inactive in CD8+ T cells when under stimulation [65]. In contrast, NFAT retains more lasting activity [66]. It can be seen that NFAT has a major role in the negative regulation that induces genes related to exhaustion or dysfunction, such as TOX [30, 33–35, 66–68].

Furthermore, there are two downstream targets of NFAT, which are the orphan nuclear receptor NR4A family and TOX family. Evidence suggests that they cooperate with NFAT to drive T cell exhaustion [30, 33–35, 66–68]. Previous studies have found that CD8+ TILs with the depletion of all the three NR4A family members, TOX, or TOX2 showed a powerful anti-tumor response [30, 33, 41]. From a mechanical perspective, the depletion of NR4A or TOX/TOX2 prevents some chromatin and transcription changes characterized by exhaustion and partially saves the effector function of TILs [67].

### BATF promotes transformation from Tpro to Teff and avoid Tex

BATF is an essential transcription factor in the differentiation of Tpro into Teff [49]. In Teff, a large number of motifs bound by BATF are enriched, and BATF is bound to a large number of enhancer regions necessary for the differentiation of Tpro into Teff [49].

In mouse tumor models, the overexpression of BATF in CD8+ CAR-T cells significantly promoted the survival and proliferation of TILs, enhanced the ability of CAR-TILs to produce cytokines and granzyme after stimulation, and inhibited the expression of the cell surface receptors and exhausted-specific transcription factor TOX [50]. IRF4 is the most important partner of BATF and can promote T cell exhaustion [69]. Overexpression of BATF induces IRF4 to be more inclined to combine with BATF at the AP-1–IRF composite element(AICE) sites with higher affinity, thus inducing the differentiation of Teff by opening the associated chromatin-accessible regions [50]. Like NFAT, the overexpressed BATF and its partner IRF4 work in conjunction against the development of Tex.

Because of its high cellular plasticity, Tpro are considered to be the greatest potential targets for the cellular immunotherapy of tumors and chronic inflammation. Previous studies have shown that anti-PD-L1 treatment can only enhance the self-replication of Tpro and is unable to guide them to differentiate into Teff [51]. IL-21 secreted by CD4+ T cells is a necessary factor for anti-PD-L1 to guide Tpro to differentiate into Teff [51, 70]. However, the helper function of CD4+ T cells is out of balance and cannot provide help for CD8+ T cells when facing chronic infections, such as HIV [71, 72]. BATF is considered the main downstream transcription factor of the IL-21 signaling pathway [73]. Therefore, editing T cells with BATF as the target can bypass the demand for IL-21, directly block the possibility of Tpro differentiating into Tex, and promote Tpro differentiate into Teff. In doing so, the ability of T cells to resist viruses and kill cancer cells is enhanced.

By classifying the enhancer regions with different chromatin accessibility in Tpro, Teff, and Tex and analyzing their transcription factor binding, it was found that the motifs of BATF were significantly enriched in the common enhancer regions of Tpro and Teff, but not in Tex [49]. In addition, BATF regulates the enhancer activity by regulating chromatin accessibility in the enhancer regions. Most of these enhancers are located in genes related to differentiation to Teff, such as Cx3cr1, Gzmb, Ifng, etc [49]. It suggested that BATF played an important role in regulating the direction of Teff differentiation and avoiding T cell exhaustion.

In summary, the high expression of BATF promotes the effective anti-tumor response in CD8+ T cells. Overexpression of BATF promotes CD8+ T cells proliferation in tumors and secretes a large number of cytokines which can partially reverse T cell exhaustion. Furthermore, BATF regulates the accessibility of enhancers to promote the transformation of Tpro into Teff and avoid Tex. From a therapeutic perspective, BATF overexpression in CAR-T cells benefits both short-term and long-term anti-tumor responses, because it also promotes the formation of long-lived memory T cells which can control tumor recurrence [50]. Future research should determine whether BATF cooperates with other TFs in adjusting these different enhanced sub-landscapes.

### NR4A is an essential regulatory factor to induce Tex

NR4A1 (Nur77) is a member of the NR4A subfamily of orphan nuclear receptors in the steroid thyroid receptor family. The other two members in this subfamily are NR4A2 (Nurr1) and NR4A3 (Nor1) [74]. NR4A1 was originally believed to be encoded by a growth factor-induced gene. It is a well-known TCR-induced target gene in thymocytes [75]. NR4A1 is often overexpressed in various cancer cells, including lung cancer, prostate cancer, breast cancer, and colon cancer, and is considered to represent the survival signal that mediates the growth of cancer cells [76]. In immune cells, the function of NR4A1 is just the opposite and is considered to be related to the programmed death of T lymphocytes and B lymphocytes [77, 78].

In the tumorigenesis and chronic infection model, the expression of transcription factor NR4A1 was up-regulated in Tex. The overexpression of NR4A1 decreased the expression of the genes, which expressed IL-2, and blocked the production of cytokines IL-2 and IFN-γ [79, 80]. However, knocking out NR4A1 in T cells enhances the secretion of cytokines [30, 41]. In the tumor model, the expression of exhausted markers PD-1 and TIM-3 decreased significantly in the NR4A1-deleted TILs. NR4A1-deleted TILs are capable of controlling tumor growth more effectively and reducing the size of a tumor’s mass [41]. It is seen that the overexpression of NR4A1 can promote the occurrence of T cell exhaustion and deleted NR4A1 restrain the effector function of T cells.

Using ATAC-seq to assess the molecular mechanism for regulating T cell exhaustion, researchers working on two separate studies put forward the same molecular regulation pathway [30, 41]. Evidence shows that the binding sites of NR4A1 and c-Jun (the subunit of transcription factor AP-1) overlap significantly, and the greatest binding site of NR4A1 is the AP-1 consensus sequence, which indicates that NR4A1 induces T cell tolerance by antagonizing AP-1-mediated gene expression [29]. NR4A1 is preferentially recruited to the binding site of transcription factor AP-1, and then inhibits Teff by inhibiting AP-1 function [30, 41]. As a transcription factor, NR4A family members regulate the expression of a series of receptors related to T cell immunosuppression by influencing the NFAT-NR4A signal axis, thus regulating T cell exhaustion [30].

The NR4A family members NR4A1 and NR4A2 are up-regulated in CD8+ TILs of human melanoma and metastatic melanoma [30, 81–83]. The expression of these proteins is closely related to the expression of PD-1 and TIM-3. The chromatin of CD8 + PD-1^high^TILs in human melanoma and non-small cell lung cancer is rich in NR4A binding motifs. It indicates that the NR4A transcription factor family is involved in regulating T cell exhaustion [84]. In the analysis of gene expression and chromatin accessibility atlas, the expression of inhibitory receptors, such as PD-1, TIM-3, TIGIT, 2B4/CD244, and CD38, was down-regulated in the triple knockout (NR4A1-/-NR4A2-/-NR4A3-/-) CD8+ TILs [84]. Therefore, the NR4A transcription factor family is an indispensable regulatory factor in the process of inducing Tex, and the potential regulatory mechanism at work may begin with NFAT initiating T cell exhaustion by up-regulating the expression of NR4A2 and NR4A3 (and other related transcription factors). Subsequently, NR4A2 and NR4A3 (and other factors) are activated independently or in combination with NFAT to maintain T cell exhaustion [40]. It suggests that NR4A could be a key therapeutic target for reversing T cell exhaustion in tumors and chronic infections.

In summary, ATAC-seq provides researchers with novel and comprehensive insights into the epigenetic regulation mechanism of T cell exhaustion in tumorigenesis and chronic viral infections. In addition to the well-known biological targets related to T cell exhaustion such as PD-1, T-bet, Eomes, and TCF1 [21], different transcription factors such as TOX, NFAT, BATF, and NR4A can be used to provide further insights, especially for the complex interaction among different transcription factors. With the development of ATAC-seq technology, the use of epigenetic information to understand the molecular pathways driving disease-related cell states greatly facilitates researches into the potential epigenetic regulation mechanism of T cell exhaustion. However, there is a need for further studies on Tex differentiation by regulatory factors and signaling pathways. We believe that future research will reveal more Tex-specific biological targets in the future, and researchers will gain a deeper understanding and further insights in this field. Future studies on these regulatory pathways may result in new therapeutic interventions that have synergistic effects with PD-1 blocking in cancer.

## ATAC-seq complements the molecular dynamic changes after anti-PD-1/PD-L1

The discovery of the immune checkpoint inhibitors was an important milestone in understanding and reversing T cell exhaustion [3, 85]. The expression of various inhibitory cell surface receptors increases in Tex, including PD-1 [86], LAG3 [87], TIM3, and CTLA-4 [86]. Earlier research uncovered two immune checkpoints, CTLA-4 and PD-1 [88]. Antibodies against these pathways have shown great achievement reversing T cell exhaustion. In particular, the anti-PD1 antibody is thought to restore the activity of Tex by preventing the attenuation of PD-1-mediated proximal TCR cascade [89, 90]. Anti-PD-1 blocking will also lead to changes in metabolic reprogramming, which is the intermediary of T cell rejuvenation [91]. Relevant studies have reviewed the basic mechanisms of immune checkpoint inhibitors [3, 92–98] and its therapeutic effect [99–101], but our study focuses on the molecular dynamic changes and the epigenetic regulation mechanism of T cell exhaustion, after anti-PD-1 was discovered through ATAC-seq.

The −23.8 kb region of the inhibitory receptor PDCD1 gene locus shows obvious chromatin accessibility in Tex. This can be used as an enhancer for maintaining high-level PD-1 expression through the CRISPR-Cas9 technique. In all the genes within 1.5 Mb of the PDCD1 locus, only PD-1 mRNA expression was significantly decreased due to the deletion of −23.8 kb ChAR [17]. This indicates that −23.8 kb ChAR as an enhancer is indispensable for maintaining a high level of PD-1 expression in exhausted CD8+ T cells. At the same time, the TF footprints of Tex were determined by ATAC-seq cleavage sites, where the main enriched motifs are Sox3, T-bet (encoded by Tbx21), and the retinoic acid receptor (RAR) [17, 102, 103].

Interestingly, the anti-PD-L1 treatment of Tex can induce the recovery of genes expression related to effector function of Teff [17, 57]. However, such treatment will not cause the chromatin accessibility of Tex to recover in cancer [40]. This is also observed in chronic viral infections [7]. In fact, Tex in tumor or virus models and human patients are not always easily reactivated [7, 83, 104]. In addition, PD-1/PD-L1 participates in the proximal signaling pathway, which is largely assumed to inhibit TCR. For example, this may occur by increasing phosphatase activity and inducing degradation of TCR by the transcription of E3 ligase [105, 106]. Therefore, the use of blocking antibodies to interrupt the interaction of inhibitory signals PD-1/PD-L1 may not have a significant impact on the chromatin accessibility spectrum, especially in those areas where chromatin accessibility is stably maintained after activation [18, 107]. In addition, although progenitor Tex may be reversible, the hyporesponsive state is partly imposed through epigenetic mechanisms, such as DNA methylation, in terminal Tex [108]. These mechanisms are generally difficult to reverse [7, 109]. Although PD-1/PD-L1 antibody therapy can temporarily restore the function of Tex, it cannot produce a lasting restoration due to the epigenetic irreversibility of T cells in the terminal stage of exhaustion [7, 110]. As such, these Tex cannot be transformed into true cytotoxic Teff. The epigenetic stability of Tex has been partially locked in the early stage of infection by Tox, a core transcription factor specific to Tex [33–35, 54]. The most probable explanation for above is that anti-PD-1 alone may be insufficient for completely reversing Tex. Thus, the treatment cannot completely save or reverse the characteristics of exhausted-specific chromatin and the exhausted-specific transcription process of Tex [7, 16, 17, 57]. The benefits of immune checkpoint inhibitors are derived from the short-term recovery of Tex but not the permanent change of their status. This also provides an explanation as to why anti-PD-1 tumor immunotherapy has a good curative effect at the initial stage but is weak at the follow-up stage. Therefore, the combined treatment of anti-PD-1 and blocking the epigenetic exhaustion-specific targets may reverse Tex to a greater extent. For example, Co-blockade of LAG-3/PD-1 or TIM-3/PD-1 pathways in chronic LCMV infection has already showed a strong and collaborative reversal of T cell exhaustion with similar results in tumor and other models of infection [111, 112]. As a consequence, Tex in cancer requires further in-depth epigenetic molecular characterization.

## Conclusions and perspectives

Presently, despite the limitations of the low efficacy of single-drug treatment and the lack of benefits for some patients, immunotherapy is still a promising strategy for cancer treatment. The enhancement of immunotherapy efficacy through reversing T cell exhaustion is one of the crucial tasks for future research. This review summarizes the main changes among Tex and Teff/Tmem/Tpro or between progenitor Tex and terminal Tex in chromatin accessibility and epigenetic characteristics observed by using ATAC-seq. There is now a new and more comprehensive view on the potential epigenetic regulation mechanism of Tex, especially in regard to the complex interaction between different transcription factors. This emphasizes the potential therapeutic targets that can be used to restore the effector function of CD8+ T cells. Finally, this study explains why immune checkpoint blocking can only temporarily restore the effector function of Tex. The reason lies in the irreversible changes of the epigenetics trait of Tex. Although the mechanism of Tex has been well understood and new treatment opportunities have been found, there remain many key issues that have yet to be solved. For example, there is a question as to whether Tex can be completely reversed, and our present understanding of the long-lived memory of T cells is insufficient. We speculate that the answer may be related to the lineage relationship among Tex, Teff, and Tmem, the population dynamics of Tex after reprogramming, and the different responses of T cell subsets at different stages to the treatment of immune checkpoint inhibitors. Therefore, researchers should consider conducting a large number of prospective experiments to explore when immunotherapy should be combined with other therapies, such as targeted drugs, and to verify relevant targets in multiple cancer types. Some studies have already begun to predict the prognosis of cancer patients according to the specific chromatin accessibility [113]. In the future, ATAC-seq will be found expanded application in epigenetics researches, which will help enhance our understanding of the molecular state of diseases.

## Supplementary information

Supplemental Table 1
