# Supplementary material for: Application of ATAC-seq in tumor-specific T cell exhaustion
Source: Cancer Gene Ther. 2022 Jul 6;30(1):1–10. doi: 10.1038/s41417-022-00495-w (PMC9842510; doi:10.1038/s41417-022-00495-w)
Supplement: Supplementary file 1 — Supplemental Table 1 [file 41417_2022_495_MOESM1_ESM.docx]

Supplemental Table1: Progenitor Tex and terminal Tex possess distinct epigenetic and transcriptional states.

| **progenitor Tex** | | **terminal Tex** | |
| --- | --- | --- | --- |
| transcription factors | TCF-1 and BATF | transcription factors | Id2, Runx1 and Prdm1 |
| costimulatory molecules | ICOs and Tnfsf14 | co-inhibitory receptors | TIM-3,ENTPD1 and CD244 |
| memory  molecules | IL7R, Id3 and Satb1 | effector molecules | Gzmb, Prf1 and Ifng |
| cytokines | TNF and IL-2 |  |  |

It has been found that genes encoding cytokines (TNF and IL-2), costimulatory molecules (ICOs and Tnfsf14), survival/memory molecules (IL7R, Id3 and Satb1), chemotactic receptor CXCR5, and transcription factors TCF-1 and BATF, in progenitor Tex, had greater chromatin accessibility and higher transcript level(16,49,50). In contrast with progenitor Tex, genes encoding co-inhibitory receptors (TIM-3, Entpd1 and CD244), effector molecules (Gzmb, Prf1 and Ifng), and transcription factors (Id2, Runx1 and Prdm1) had more chromatin accessibility and expression in terminal Tex (16,44,45).
